# Guidance on Energy Intake Based on Resting Energy Expenditure and Physical Activity: Effective for Reducing Body Weight in Patients with Obesity

**DOI:** 10.3390/nu17020202

**Published:** 2025-01-07

**Authors:** Tomoko Handa, Takeshi Onoue, Ryutaro Maeda, Keigo Mizutani, Koji Suzuki, Tomoko Kobayashi, Takashi Miyata, Mariko Sugiyama, Daisuke Hagiwara, Shintaro Iwama, Hidetaka Suga, Ryoichi Banno, Hiroshi Arima

**Affiliations:** 1 Department of Endocrinology and Diabetes, Nagoya University Graduate School of Medicine, Nagoya 466-8550, Japan; 2 Department of Clinical Research Education, Nagoya University Graduate School of Medicine, Nagoya 466-8550, Japan; 3 Research Center of Health, Physical Fitness and Sports, Nagoya University, Nagoya 464-8601, Japan

**Keywords:** resting energy expenditure, weight loss, energy intake, energy expenditure, hunger, weight loss program, caloric restriction, physical activity, weight management

## Abstract

Objective: In treating obesity, energy intake control is essential to avoid exceeding energy expenditure. However, excessive restriction of energy intake often leads to resting energy expenditure (REE) reduction, increasing hunger and making weight loss difficult. This study aimed to investigate whether providing nutritional guidance that considers energy expenditure based on the regular evaluation of REE and physical activity could effectively reduce body weight (BW) in patients with obesity. Methods: A single-arm, prospective interventional study was conducted on 20 patients with obesity (body mass index ≥ 25 kg/m^2^) at the Nagoya University Hospital for 24 weeks. REE and physical activity were regularly assessed, and the recommended energy intake was adjusted based on the values. The primary outcome was the change in BW, and the secondary outcomes included changes in REE and hunger ratings, which were assessed using a visual analog scale. Results: Eighteen participants completed the study, demonstrating a significant reduction in BW after 24 weeks (−5.34 ± 6.76%, *p* < 0.0001). No significant changes were observed in REE or hunger ratings. No adverse events were reported throughout the study period. Conclusions: Guidance on energy intake based on REE and physical activity was effective for reducing BW in patients with obesity without decreasing REE or increasing hunger. This approach may reduce the burden on patients with obesity while losing BW.

## 1. Introduction

Globally, obesity, with increasing prevalence despite extensive efforts to combat it, is one of the most pressing public health challenges [1]. Although pharmacotherapy and surgical interventions are currently available as treatment options for obesity, dietary management remains fundamental [2]. Moreover, ways to set up the appropriate energy intake for weight loss are inconsistent among guidelines despite most dietary interventions prescribing energy restriction for weight loss [3,4,5,6,7]. Furthermore, these guidelines do not address individual variations in energy expenditure (EE).

Energy balance is regulated by energy intake and EE, with the latter consisting of resting EE (REE, approximately 60%), physical activity (approximately 30%), and diet-induced thermogenesis (approximately 10%) [8,9]. When energy intake consistently exceeds the EE, weight gain occurs, leading to obesity. To treat obesity, energy intake must be controlled so that it does not exceed the EE, which can be achieved by either reducing the energy intake or increasing the EE.

To maintain homeostasis of energy balance, the body adjusts REE in response to energy intake changes. During weight loss, REE reduction often exceeds what is expected based on the reduction in the free fat mass (FFM) and fat mass (FM), a phenomenon known as metabolic adaptation or adaptive thermogenesis [10,11]. This response is considered our body’s biological drive to regain the body mass we lose to survive [12,13]. The mechanisms underlying this response were associated with reductions in the levels of thyroid hormones [14,15], leptin [14,16], catecholamines [15], and insulin [11], which occur during weight loss. Moreover, dietary restriction and weight loss are often accompanied by increased hunger [17,18,19,20,21], which could lead to greater energy intake, potentially making it more difficult to achieve weight loss [20] and contributing to weight regain [21,22]. Therefore, suppressing the increase in hunger during weight loss is also important for successful weight management.

Previously, we showed that in patients with obesity, the change in REE one week after the start of weight loss was significantly correlated with the energy intake/EE ratio set at the start of weight loss and that even if REE decreases after the first week, this ratio also significantly correlated with changes in REE one more week later [23]. Furthermore, our data showed that setting the energy intake/EE ratio to 80–90% one week after the start of weight loss allowed the REE to recover to its baseline levels [23].

In this study, we examined whether nutritional guidance based on EE, which was regularly calculated from the evaluation of REE and physical activity levels, could be effective for weight loss and prevent decreases in REE and increases in hunger.

## 2. Materials and Methods

### 2.1. Study Design

This 24-week, open-label, single-armed prospective study was conducted at the Nagoya University Hospital in Japan. The study protocol was approved by the ethics committee of the Nagoya University Graduate School of Medicine (No. 2021-0021). The study was conducted in accordance with the ethical principles of the Declaration of Helsinki. After the participants were informed of the study’s purpose and potential risks and benefits, they provided written consent to participate. The trial is registered in the University Hospital Medical Information Network (UMIN) Clinical Trials Registry (UMIN000044385).

### 2.2. Patients

We recruited patients with obesity and one or more obesity-related disorders from the Nagoya University Hospital. This study defined obesity based on the Japan Society for the Study of Obesity (JASSO)’s definition, i.e., a body mass index (BMI) of ≥25 kg/m^2^ [3], which includes the World Health Organization’s BMI range for overweight (25.0–29.9 kg/m^2^) [24]. Obesity-related disorders were diagnosed on the basis of the JASSO guidelines [3]. The inclusion and exclusion criteria for the study are detailed in Table 1.

### 2.3. Registration

Participants who qualified for the above criteria and visited the Nagoya University Hospital between 1 June 2021, and 18 April 2024, were eligible for recruitment.

### 2.4. Intervention

**Figure 1:**
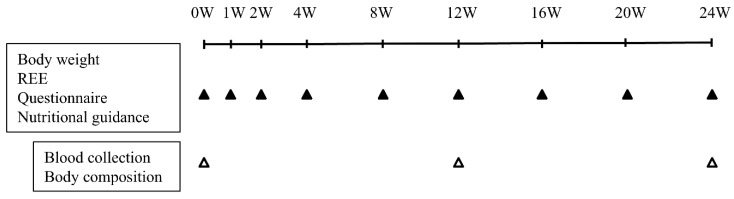
Study schedule. REE, resting energy expenditure. Black triangles (▲): Time points for body weight, REE (Resting Energy Expenditure), questionnaires, and nutritional guidance. White triangles (△): Time points for blood collection and body composition.

Patients who were attending our hospital and met the criteria for this study received an explanation of the study, and informed consent was obtained. Those who gave consent participated in “a weight loss program by setting energy intake considering changes in the REE”, which involved periodic evaluation of REE, physical activity, and body composition as well as nutritional guidance on the appropriate diet based on EE. During the study, blood samples were collected, and dietary intake was monitored. The study schedule is presented in Figure 1. “A weight loss program by setting energy intake considering changes in the REE” consists of four key steps (Table 2).

The details of our weight loss program are as follows:

First, EE was calculated as the sum of REE, EE from physical activity was estimated using the international physical activity questionnaire (IPAQ), and 10% of the total EE was estimated as diet-induced thermogenesis. The IPAQ was chosen for creating a weight loss program that is more feasible and practical in clinical settings, facilitating EE estimation from physical activity in a cost-effective and straightforward manner while maintaining reasonable accuracy [25].

Second, the recommended energy intake is determined by considering both the estimated EE and the energy deficit required for weight loss. JASSO sets the target weight loss after 3–6 months as at least 3% of the current body weight (BW) for patients with obesity (25 kg/m^2^ ≤ BMI < 35 kg/m^2^) and 5–10% of the current BW for patients with severe obesity (BMI ≥ 35 kg/m^2^) [3]. In this study, the target weight loss after 6 months was set as 10%. The detailed algorithm is described separately (Supplementary Information).

Third, the recommended intake was explained through nutritional guidance with specific menu examples using the 2020 edition of the Standard Tables of Food Composition in Japan (8th revision) [26] and photographs to enhance clarity. Patients were instructed to use an application called Calomeal [27]. This application allows users to photograph their meals with a smartphone camera, which facilitates the recording and management of nutrient and energy intake. In this app, automatic artificial intelligence analysis enables the calculation of calories, carbohydrates, sugars, proteins, fats, sodium, and dietary fibers.

Finally, the above process was repeated at the time of participation (week 0) and visits during the intervention period (weeks 1, 2, 4, 8, 12, 16, and 20).

### 2.5. Data Collection

The following measurements were conducted at the Nagoya University Hospital.

#### 2.5.1. BW and Composition

BW was measured using a calibrated digital scale (to the nearest 0.1 kg; PW-650A, TANITA Corp., Tokyo, Japan), and body composition was measured using a bioelectrical impedance analysis device, InBody (S-10, InBody Japan Inc., Tokyo, Japan). Both measurements were taken in the fasting state immediately after the participants had voided in the morning. Specifically, BW was measured at weeks 0, 1, 2, 4, 8, 12, 16, 20, and 24, and body composition was measured at weeks 0, 12, and 24.

#### 2.5.2. REE

REE was measured using a handheld indirect calorimetry (MedGem, HealtheTech, Inc., Golden, CO, USA) [28,29,30,31,32]. All tests were conducted between 8:00 and 9:00 AM, after a fast for at least 12 h. Testing was performed in a quiet, softly lit, well-ventilated room, with the temperature maintained between 22 °C and 24 °C. Patients were instructed to rest in a seated position for 10 min before measurement. The patients sat upright and wore a nose clip, and a disposable mouthpiece was placed in the mouth. Then, they were asked to remain still and breathe via the mouthpiece during the test (approximately 10 min). The first 2 min were eliminated as patient acclimatization to the instrument, and a steady-state VO_2_ measurement was obtained during the next 3–8 min using a rolling boxcar methodology on reiterative sets of VO_2_ in 30 breaths. The analysis was conducted at the time of participation (week 0) and during intervention visits (weeks 1, 2, 4, 8, 12, 16, 20, and 24).

#### 2.5.3. Physical Activity

IPAQ, one of the questionnaires used to evaluate physical activity, was used, and the obtained data were analyzed to estimate the average daily physical activity [33]. IPAQ is a convenient and useful method for assessing physical activity that allows for international comparisons. It is comparable or superior to traditional physical activity questionnaires in terms of reliability and validity [25]. The data were analyzed at the time of participation (week 0) and during each intervention visit (weeks 1, 2, 4, 8, 12, 16, 20, and 24).

#### 2.5.4. Diet-Induced Thermogenesis

Diet-induced thermogenesis, which is 6–10% of energy intake [8,9], was estimated as 10% of the total EE, as its actual measurement is difficult.

#### 2.5.5. Clinical Parameters

Clinical parameters such as blood pressure, blood biochemistry results (collected at weeks 0, 12, and 24), medical history, and therapy during hospitalization were collected from electronic medical records.

### 2.6. Outcomes

The primary outcome was the change in BW. Secondary outcomes included changes in REE, hunger scale, body composition, BMI, HbA1c levels, lipid profiles, various blood test items, blood pressure, energy intake, and adverse events (safety). Hunger was assessed at weeks 0, 1, 2, 4, 8, 12, 16, 20, and 24 using a visual analog scale (VAS), where participants were asked to rate their levels of hunger on a scale ranging from 0 (not hungry at all) to 10 (never been more hungry) [34]. Dietary intake was assessed using the brief-type self-administered diet history questionnaire (BDHQ), a validated tool that captures the frequency and amount of food and beverage consumption over the past month [35,36]. BDHQ data were analyzed at weeks 0, 12, and 24 to estimate the daily nutrient intake.

### 2.7. Sample Size

Based on the results of a previous clinical trial that examined the effect of weight loss on patients who are obese and overweight, we estimated that the standard deviation of the change in BW (%) before and after the intervention would be approximately 2.0% [37] and that the 95% confidence interval of the change in BW (%) could be evaluated with an accuracy of approximately ±1.8 (%) if approximately 20 cases could be recruited in this exploratory study.

### 2.8. Statistical Analysis

Continuous variables are presented as median (min–max) and categorical variables as numbers (percentages). A linear mixed model, including the treatment period as a fixed effect, was used to compare changes in BW, REE, hunger scale, body composition, BMI, HbA1c level, lipid profiles, various blood test items, blood pressure, energy intake, and adverse events (safety) from baseline. JMP Pro version 15.1.0 (SAS Institute Inc., Cary, NC, USA) was used for all statistical analyses. A *p* value of <0.05 was considered significant.

## 3. Results

**Figure 2:**
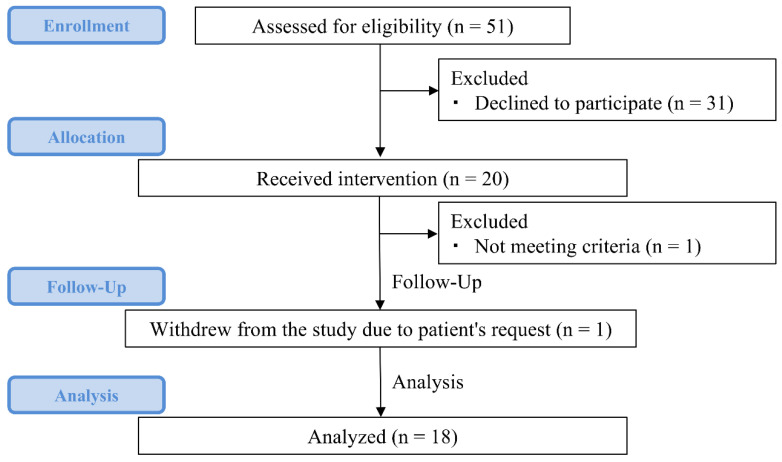
CONSORT flow diagram.

Figure 2 shows the CONSORT flow diagram of the study. In our hospital, 51 candidates were assessed for eligibility for this study, and 20 patients were enrolled. Of the 20 patients recruited for the study, 1 did not meet the inclusion criteria, and another withdrew, resulting in a total of 18 patients completing the study.

Table 3 shows the baseline characteristics of the patients. Six patients were men and 12 were women, with a median age of 52.5 years (range: 26.0–63.0) and a median BMI of 32.8 kg/m^2^ (26.4–57.6). Approximately 61.1% of the patients presented with type 2 diabetes, 66.7% with hypertension, and 61.1% with dyslipidemia.

**Figure 3:**
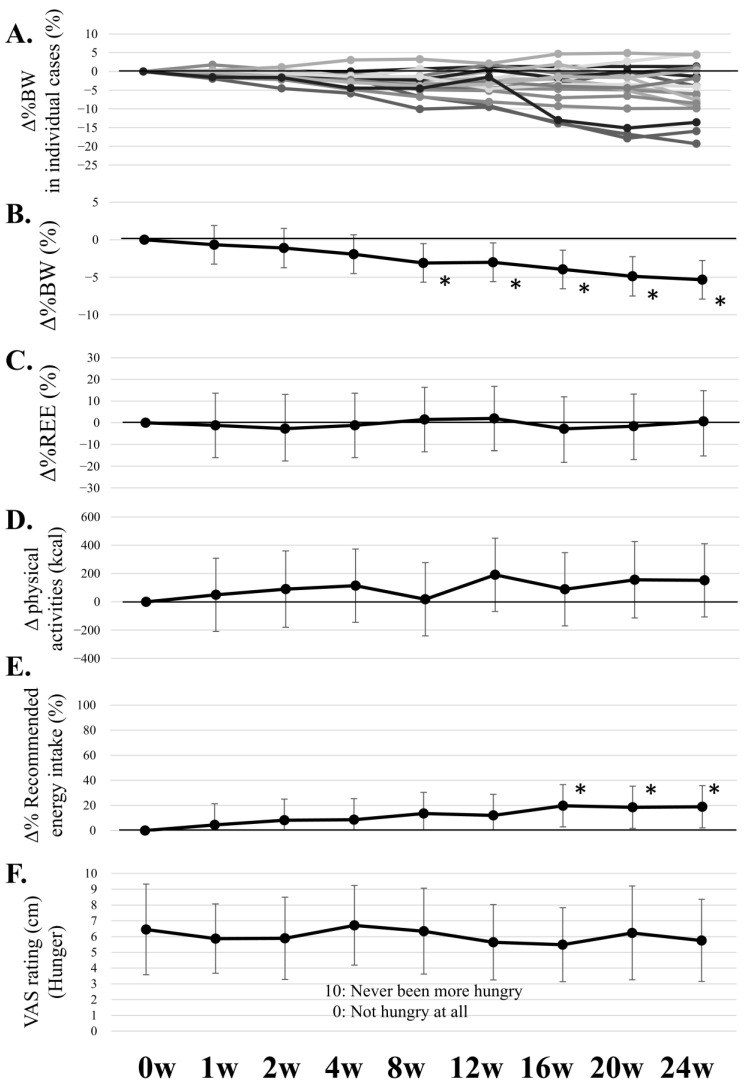
The changes in body weight, REE, physical activities, recommended energy intake, and VAS rating (hunger) during the weight loss program. The bars show the 95% confidence intervals (Δ% BW, Δ% REE, Δ physical activities, and Δ% recommended energy intake) or standard deviation (VAS rating). * *p* < 0.05 vs. baseline value. BW, body weight; REE, resting energy expenditure; VAS, visual analog scale. (**A**) Individual changes in %BW, with each line representing an individual case. (**B**) Mean changes in %BW. (**C**) Mean changes in %REE. (**D**) Mean changes in physical activities (kcal). (**E**) Mean changes in % recommended energy intake. (**F**) VAS ratings for hunger (cm).

Figure 3 presents the changes in BW, REE, physical activities, recommended energy intake, and VAS rating (hunger) during the weight loss program. BW decreased by ≥10% in three patients, 5–10% in six, and 0–5% in four, whereas it increased by 0–5% in five. In total, BW decreased significantly (−5.34% ± 6.76%, *p* < 0.0001) after 24 weeks. The recommended energy intake significantly increased at 16, 20, and 24 weeks. In contrast, no significant changes were observed in REE or hunger ratings throughout the program.

Table 4 shows the changes in metabolic outcomes, blood pressure, and various blood test items among all patients. Compared to the baseline values, BMI decreased by −1.36 kg/m^2^ at 12 weeks (95% (CI), −2.44 to −0.29, *p* = 0.013) and −2.12 kg/m^2^ at 24 weeks (95% CI, −3.19 to −1.04, *p* = 0.0001). In addition, body FM decreased by −3.39 kg at 12 weeks (95% CI, −6.22 to −0.56, *p* = 0.0172) and −6.91 kg at 24 weeks (95% CI, −9.75 to −4.09 kg, *p* < 0.0001). However, no significant changes were observed in lean body mass, muscle mass, or skeletal muscle mass throughout the program. The estimated energy intake, as assessed by BDHQ, decreased by −316.60 kcal/day at 12 weeks (95% CI, −595.13 to −38.06, *p* = 0.0242) and −333.51 kcal/day at 24 weeks (95% CI, −612.05 to −54.98, *p* = 0.0173) compared to the values at week 0. In addition, triglyceride decreased by −0.33 mg/dL at 12 weeks (95% CI, −0.63 to −0.03, *p* = 0.0283) and −0.36 mg/dL at 24 weeks (95% CI, −0.66 to −0.06, *p* = 0.0160). Moreover, the aspartate transaminase levels decreased by −8.89 (95% CI, −17.37 to −0.40, *p* = 0.0391), alanine transaminase by −16.72 (95% CI, −29.83 to −3.61, *p* = 0.0110), and gamma-glutamyl transpeptidase by −9.22 (95% CI, −17.05 to −1.39, *p* = 0.0193) at 24 weeks.

No adverse events were observed during the study period.

## 4. Discussion

In this study, REE and physical activity were regularly evaluated in patients with obesity, and they received nutritional guidance based on these measurements. Our findings demonstrated that setting the energy intake based on the EE led to a decrease in estimated energy intake assessed by BDHQ and was effective for reducing BW in patients with obesity without inducing reductions in REE or increases in hunger.

Obesity guidelines recommend reducing the energy intake by 500 [4], 600 [38], 500–750 [5,39], and 500–1000 kcal per day [6,7,40,41], or by 30% [5] for weight loss. For more specific targets, the daily energy intake is often set at 1200–1500 kcal for women and 1500–1800 kcal for men [4,5]. In addition, JASSO recommends setting the energy intake based on the goal BW and an energy coefficient: the goal BW is defined as the weight corresponding to a BMI of 22 for individuals aged <65 years and a BMI of 22–25 for those aged ≥65 years, whereas the energy coefficient is set at 25 for individuals with obesity (BMI 25–35), and 20–25 for those with severe obesity (BMI ≥ 35) [3]. Conversely, EE varies according to age [42], sex [43], and body composition (particularly FFM) [44,45], even among individuals of the same height. Therefore, when setting the recommended energy intake based on the methods described above, the caloric setting may not be appropriate, potentially affecting the effectiveness and safety of weight loss programs. In this study, REE was evaluated using indirect calorimetry and physical activity with a questionnaire, and the recommended energy intake was set based on the estimated EE. This approach allowed for energy intake adjustment tailored to individual patients and enabled the regular adjustment of energy intake in accordance with changes in REE or physical activity over the course of weight loss. In addition, the regular monitoring of clinical parameters and symptoms by the attending or consulting physician ensured malnutrition did not occur during the intervention.

The persistence of REE reduction during weight loss, known as metabolic adaptation (or adaptive thermogenesis), is an important factor that makes weight maintenance challenging in patients with obesity [46,47,48,49]. Conversely, we previously reported that even when BW decreases and REE is reduced, setting the energy intake to match the EE could minimize metabolic adaptation and promote REE recovery [23]. In the present study, REE was regularly measured during the weight loss process, and the appropriate energy intake was adjusted. This approach likely helped prevent the decrease in REE. Another weight loss program that can potentially help suppress metabolic adaptation is intermittent energy restriction (IER). IER is a weight loss method that alternates between periods of significant caloric restriction and periods of normal caloric intake. Reportedly, IER can achieve long-term weight loss while preventing REE reduction associated with caloric restriction [50,51]. However, IER has the potential risk of rebound weight gain due to compensatory behaviors. Generally, energy intake tends to increase following periods of energy restriction or fasting [52,53,54] thus diminishing the weight loss effect.

Weight loss is often associated with increased hunger [19], which was reported to heighten negative emotions such as tension, anger, and loneliness [55,56,57], and be associated with increased stress levels [58]. In this study, despite undergoing weight loss, the hunger ratings on the hunger scale did not increase (Figure 2). Thus, setting appropriate energy intake in this weight loss program, which could avoid excessive dietary restriction, may prevent an increase in hunger.

In this study, patients used the dietary recording app “Calomeal”, where they managed their diet by taking pictures of their meals with a smartphone camera. Paper recording of diet can be burdensome for patients and makes it difficult for nutritionists to accurately assess nutrient intake [59]. In contrast, recording through an app with photos taken by a camera can reduce these problems [60,61] and is generally preferred by users [62,63]. These features of dietary recording apps may help patients adjust their meals to align with their recommended energy intake and enable physicians to provide personalized feedback more efficiently.

This study has several limitations. First, it had a relatively small sample size, and all participants were recruited from a single center. Future studies should have a larger number of patients from multiple centers to validate the findings of this study. Second, the assessment of EE from physical activity was conducted using IPAQ. Although IPAQ is a validated and widely used questionnaire, it is susceptible to recall bias and social desirability bias because it is a self-reporting tool. Third, the study duration was 24 weeks, and the insight into whether the weight loss achieved can be sustained over the long-term remains unclear.

## 5. Conclusions

This study demonstrated the safety and effectiveness of a weight loss program for patients with obesity by regularly evaluating REE and physical activity and providing nutritional guidance based on these measurements. Energy intake guidance based on EE effectively reduced BW in patients with obesity without decreasing REE or increasing hunger. This approach may reduce the burden on patients with obesity while losing BW. Future research should focus on the program’s long-term outcomes and applicability in broader clinical settings.

## Figures and Tables

**Table 1:** Inclusion/exclusion criteria.

**Inclusion Criteria**
(1) Patients diagnosed with obesity, defined by a body mass index of 25 kg/m^2^, and at least one obesity-related disorder (2) Patients aged ≥18 and <65 years (3) Patients who have not participated in any other weight loss programs in the last 8 weeks (4) Patients who have understood the research details and provided informed consent
**Exclusion Criteria**
(1) Patients with secondary (symptomatic) obesity (2) Patients with a history of major surgery, severe trauma, severe infection disease, malignant tumors, or hyperthyroidism within 6 months (3) Patients with severe impairment in cardiorespiratory, renal, or hepatic function for whom exercise therapy is not feasible (including patients with an estimated glomerular filtration rate of <30 mL/min/1.73 m^2^) (4) Patients with severe musculoskeletal impairments for whom exercise therapy is not feasible (5) Patients who are pregnant, suspected of being pregnant, within 28 days postpartum, or currently breastfeeding (6) Patients who are judged by their physicians as unsuitable for participation in the study

**Table 2:** Four main steps of our weight loss program.

Step	Details
**1. Estimate the energy expenditure**	Total energy expenditure was calculated by combining REE; physical activity was estimated using IPAQ and adding 10% of total expenditure as diet-induced thermogenesis.
**2. Determine the recommended energy intake**	The recommended energy intake is determined using an algorithm based on the energy expenditure and the caloric deficit needed for weight loss. The target weight loss is set at 10% over 6 months.
**3. Provide nutritional counseling**	Nutritional guidance, including menu examples and photographs, is provided to support compliance. Nutrient intake is monitored through a meal recording app.
**4. Repeat the process at specific intervals**	The process was conducted at baseline (week 0) and follow-up visits (weeks 1, 2, 4, 8, 12, 16, and 20).

**Table 3:** Baseline characteristics of participants.

	Total Patients (*n* = 18)
Age (years)	52.5 (26.0–63.0)
Sex, male	6 (33.3%)
Body weight (kg)	90.9 (62.0–170.5)
BMI (kg/m^2^)	32.8 (26.4–57.6)
Waist circumference (cm) ^a^	103.0 (87.5–151.0)
Type 2 diabetes	11 (61.1%)
Hypertension	12 (66.7%)
Dyslipidemia	11 (61.1%)
Hyperuricemia	10 (55.6%)
Proteinuria	7 (38.9%)
Cardiovascular disease	0 (0%)
Cerebrovascular disease	0 (0%)
MASLD	14 (82.4%)
OSAS, OHS	3 (16.7%)
Musculoskeletal diseases ^b^	1 (5.6%)
Thyroid disease ^c^	2 (11.1%)
Menstrual disorder ^d^	1 (5.6%)
Oral steroids ^e^	1 (5.6%)
Anti-obesity drug	0 (0%)
Diuretic	2 (11.1%)
SGLT-2 inhibitor	7 (38.9%)

Data were expressed as median (min–max), or *n* (%) values. BMI, body mass index; MASLD, metabolic dysfunction-associated steatotic liver disease; OSAS, obstructive sleep apnea syndrome; OHS, obesity hypoventilation syndrome; SGLT-2 inhibitor, sodium-glucose co-transporter-2 inhibitor. ^a^ *n* = 17. One patient had missing data about waist circumference. ^b^ One patient presented with hip osteoarthritis. ^c^ Two patients had Graves’ disease (one without medication). Blood levels of free T3 and free T4 in both patients were normal throughout study. ^d^ One patient presented with polycystic ovary syndrome (PCOS). ^e^ One patient was taking prednisolone for fibromyalgia.

**Table 4:** Changes in metabolic outcomes, estimated energy intake as assessed by BDHQ, blood pressure, and various blood test items in all patients (*n* = 18).

	Changes at 12 Weeks (95% CI)	*p*-Value	Changes at 24 Weeks (95% CI)	*p*-Value
BMI (kg/m^2^)	−1.36 (−2.44 to −0.29)	**0.0013**	−2.12 (−3.19 to −1.04)	**0.0001**
Body composition				
Body fat mass (kg)	−3.39 (−6.22 to −0.56)	**0.0172**	−6.91 (−9.75 to −4.09)	<**0.0001**
Body fat percentage (%)	−2.19 (−4.41 to 0.04)	0.0542	−4.77 (−6.99 to −2.54)	<**0.0001**
Lean body mass (kg)	0.29 (−1.91 to 2.48)	0.9367	1.05 (−1.14 to 3.24)	0.4463
Muscle mass (kg)	0.15 (−1.97 to 2.28)	0.9799	0.88 (−1.25 to 3.01)	0.5407
Skeletal muscle mass (kg)	0.16 (−1.23 to 1.54)	0.9517	0.60 (−0.78 to 1.98)	0.5099
Estimated energy intake as assessed by BDHQ (kcal/day)	−316.60 (−595.13 to −38.06)	**0.0242**	−333.51 (−612.05 to −54.98)	**0.0173**
Blood pressure (mmHg)				
Systolic blood pressure	0.50 (−6.73 to 7.73)	0.9817	−6.31 (−13.69 to 1.06)	0.1011
Diastolic blood pressure	−0.31 (−6.18 to 5.55)	0.9891	−5.76 (−11.75 to 0.23)	0.0606
HbA1c (mmol/mol)	−1.86 (−5.68 to 2.08)	0.4562	−2.62 (−6.34 to 1.20)	0.2070
Triglyceride (mmol/L)	−0.33 (−0.63 to −0.03)	**0.0283**	−0.36 (−0.66 to −0.06)	**0.0160**
LDL cholesterol (mmol/L)	0.06 (−0.38 to 0.50)	0.9257	−0.11 (−0.55 to 0.33)	0.7944
AST (IU/l)	−6.44 (−14.93 to 2.04)	0.1555	−8.89 (−17.37 to −0.40)	**0.0391**
ALT (IU/l)	−9.56 (−22.67 to 3.56)	0.1772	−16.72 (−29.83 to −3.61)	**0.0110**
γ-GTP (IU/l)	−3.78 (−11.61 to 4.05)	0.4389	−9.22 (−17.05 to −1.39)	**0.0193**

BDHQ, brief-type self-administered diet history questionnaire; CI, confidence interval; BMI, body mass index; HbA1c, glycated hemoglobin; LDL, low-density lipoprotein; AST, aspartate aminotransferase; ALT, alanine aminotransferase; γ-GTP, γ-Glutamyl TransPeptidase. *p*-values < 0.05 shown in bold.

## Data Availability

The datasets used and analyzed during the current study will be made available to researchers who submit a methodologically sound proposal, approved by an independent review committee, that aligns with the aims described in their proposal. Proposals should be directed to the corresponding author.

## Supplementary Materials

The following supporting information can be downloaded at: https://www.mdpi.com/article/10.3390/nu17020202/s1, Figure S1: Algorithm for setting recommended energy intake; Figure S2: Screenshot of the dietary support application.
