# Supplementary material for: Guidance on Energy Intake Based on Resting Energy Expenditure and Physical Activity: Effective for Reducing Body Weight in Patients with Obesity
Source: Nutrients. 2025 Jan 7;17(2):202. doi: 10.3390/nu17020202 (PMC11767982; doi:10.3390/nu17020202)
Supplement: Supplementary file 1 [file nutrients-17-00202-s001.zip › nutrients-3397904-supplementary.pdf]

Supplementary Information

Algorithm for setting the recommended energy intake

The energy intake was set at 70% of the total energy expenditure at the beginning and gradually increased over time. When the degree of weight loss achievement is <0.5, that is, <5% weight reduction within 6 months, the energy intake remains at 70% of the total energy expenditure. If the weight loss achievement is between 0.5 and 0.8, the energy intake is increased to 80%. When the achievement falls between 0.8 and 1.0, the energy intake is set at 90%. Once the target weight is achieved, the energy intake is set to 100% of the total energy expenditure (Algorithm for setting recommended energy intake).

If the resting energy expenditure (REE) decreases by 10%–20% from the baseline, another 10% is added to the calculated energy intake. For example, when weight loss achievement dictates an energy intake of 70% and REE decreases by 10%–20%, the intake is adjusted to 80%. Similarly, if the REE decreases by >20% from the baseline, another 20% is added to the calculated energy intake. For instance, when weight loss achievement sets the energy intake at 70% and the REE decreases by >20%, the intake is adjusted to 90%. In all cases, the upper limit is set at 100% of the total energy expenditure.

Using this algorithm, a dietary support application was developed for calculating the target energy intake (Screenshot of the dietary support application). First, users input their current weight, initial weight at the start of weight loss, target weight after 6 months (10% reduction from baseline), the most recent REE value, and the initial REE value. Subsequently, the results of the International Physical Activity Questionnaire (IPAQ) are entered, and the recommended energy intake is automatically calculated. The calculated energy intake represents the target calories of the daily dietary intake.

Figure S1. Algorithm for setting recommended energy intake

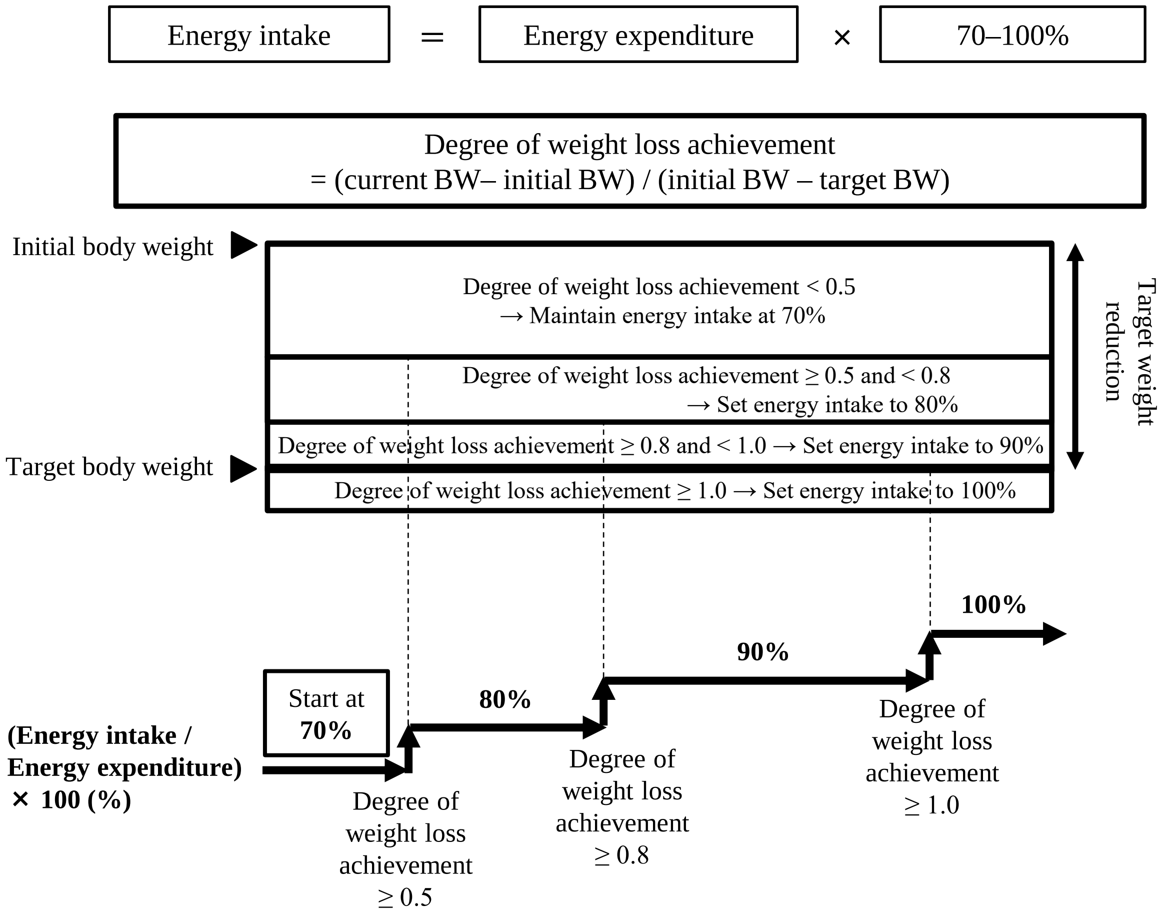

Figure S2. Screenshot of the dietary support application

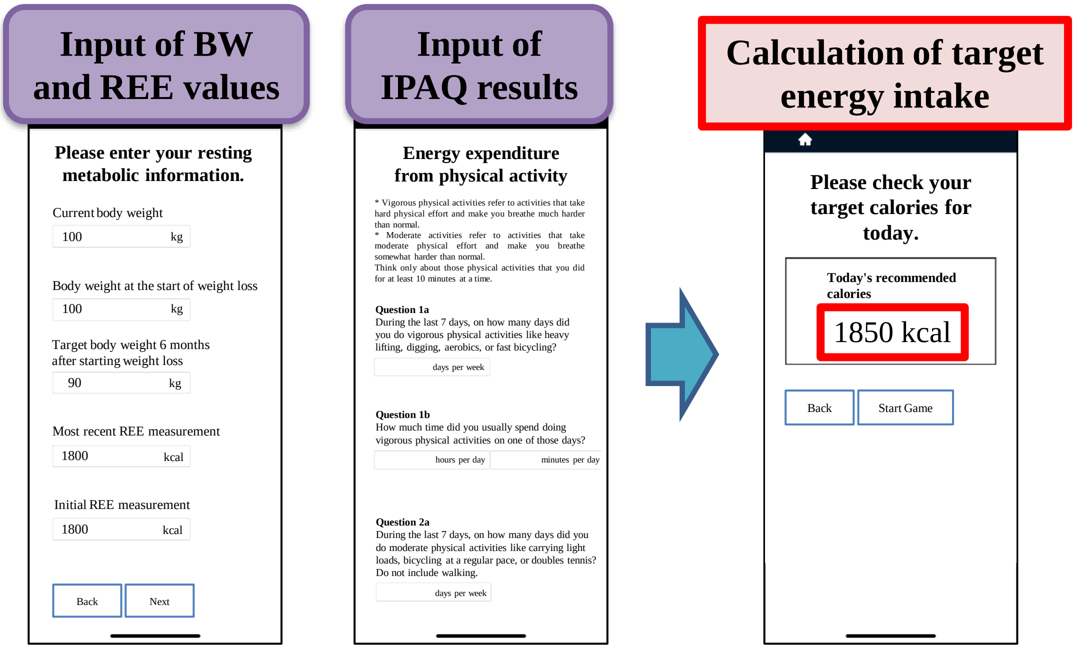

BW, body weight; REE, resting energy expenditure; IPAQ, International Physical Activity Questionnaire.
